# What difference does it make? A laboratory experiment on the effectiveness of health-oriented leadership working on-site compared to the digital working context

**DOI:** 10.1186/s12889-023-15798-2

**Published:** 2023-05-31

**Authors:** Laura Klebe, Jörg Felfe

**Affiliations:** Department of Work, Organizational and Economic Psychology, Helmut Schmidt University Hamburg, Holstenhofweg 85, 22043 Hamburg, Germany

**Keywords:** Leadership, Health-oriented leadership, Digitization, Working from home, Communication

## Abstract

**Background:**

Health-oriented leadership (HoL) represents an important workplace resource for employees. However, as opportunities to work from home increase, the question arises, whether leadership is more or less effective in digital working contexts compared to working on-site.

**Methods:**

The current research investigates, whether the effectiveness of health-oriented leadership in terms of staff care is influenced by the working context. In a laboratory experiment with a 2 (no staff care vs. staff care) x 2 (working on-site vs. digital) mixed design (N = 60), a moderating effect of the working context on the relationship between staff care and employees’ mental exhaustion, heart rate, heart rate variability, engagement and job satisfaction was tested.

**Results:**

Results uncovered positive effects of staff care on employees’ mental exhaustion and work-related attitudes in both conditions (*d* = 1.09–1.91). As expected, the results indicate that the effects on employees’ engagement (*d* = 0.65) and job satisfaction (*d* = 0.72) are weaker when working digital.

**Conclusion:**

Findings show that the effectiveness of staff care might differ between working on-site and working digital. In order to maintain the effectiveness of staff care, leaders and employees should keep regular face-to-face contact also when mainly working from home. The study ties in with research on digital leadership and leadership effectiveness, and contributes to the deeper understanding of situational contingencies of health-specific leadership during the process of digitization.

## Introduction

Within the past years, the digitization of work has experienced a boost and working from home opportunities are now a common part in the world of work. Accordingly, leaders increasingly communicate with their employees via digital media, such as e-Mail, chat or video calls. While new digital opportunities go along with many advantages for organizations and employees, such as flexibility in terms of working location and time, it is yet unknown, whether leadership can be equally effective in digital working contexts compared to working on-site.

This question also arises for health-specific leadership. The Health-oriented Leadership concept (HoL) by Franke et al. considers health-promoting self leadership (i.e., self care) and health-promoting employee leadership (i.e., staff care), which both contribute to employee health and well-being [1]. According to the HoL concept, leaders affect their employees directly through their behavior, indirectly by designing their working conditions and work tasks, and through their role model function [1]. Previous literature has proven that health-oriented leadership represents an important workplace resource for employee health, engagement and job satisfaction in traditional working contexts [1–3]. However, as previous research was mostly conducted before the pandemic and did not control for the working context, it is yet an open question, whether the same effects apply for the digital working context as for working on-site.

To date, empirical findings on leadership effectiveness in the digital context remain inconclusive. Whereas some studies suggest that leadership behaviors such as transformational leadership or LMX may be more effective in the digital than in the face-to-face context [4, 5], other research reports that transformational leadership is more effective with more face-to-face communication in contrast to digital communication [6]. Other studies do not find any difference regarding transformational, transactional or supportive leadership [7–9]. Regarding the effectiveness of health-oriented leadership in digital working environments, there are at least two plausible scenarios:

On the one hand, health-oriented leadership may be even more important for employees in digital working contexts. Working digitally, employees for example may have more difficulties in structuring their day, adhering to healthy working routines, or detaching from work in the evening. In this case, employees may be more in need of a leader who sets priorities, reminds them to take regular breaks and to finish work on time, who identifies and supports their health-related needs in terms of work organization, or who encourages them to engage in a healthy lifestyle despite spending their day in front of a screen. Employees may thus be more dependent on the guidance and support of their leaders, so that the effectiveness of leadership may even increase in the digital working context. This would be in line with previous findings, which indicate that leadership is more effective in digital working contexts [4, 5].

On the other hand, it is also conceivable that the effectiveness of health-oriented leadership is mitigated in digital working contexts without regular face-to-face contact. Verbal and non-verbal communication are core determinants of health-oriented leadership [10]. However, contact frequency, contact intensity and visual eye contact are severely restricted in digital working environments. As health and well-being are very personal and sensitive topics, it might be harder for leaders to effectively promote employee health when a personal, trusting atmosphere is not given [11].

First, it is more difficult for leaders to directly influence their employees’ stressors in the digital context [12]. In digital environments, there is often less time for informal communication, as contact frequency and intensity are reduced. Digital meetings are also often more task-focused as face-to-face meetings, so that there are less opportunities to talk about health issues [11]. Moreover, a study by Klebe et al. already showed that the effectiveness of health-oriented leadership in the digital context is also limited when communication quality is lower due to ICT hassles (e.g., low audio quality, interruptions due to system breakdowns; [33].

Second, it is questionable to what extent employees actually follow health-related recommendations of their leaders when trust and bonding is reduced [11]. For example, when leaders suggest their employees to take regular breaks or to finish work on time, their recommendations may be less binding because of less direct encounters and fewer control possibilities.

Third, also the subjective responsibility of leaders for their employees may decrease [11]. For example, it is more difficult to serve as a role model, as employees do not notice when leaders engage in self care when they are in different places (e.g., a walk during lunch break or setting up an ergonomic workplace). As leaders can also only hardly influence employees’ working conditions at home, they may feel less responsible for employee health as while working on-site.

Taken together, these factors may lead to a situation in which the efforts of leaders to engage in staff care are less effective. Employees might then experience subjective feelings of decreasing support and less health-care offered by leaders in the digital context. These considerations would support the notion that the effectiveness of health-oriented leadership may be mitigated with decreasing face-to-face contact.

The aim of the current study therefore is to analyze the effectiveness of health-oriented leadership in terms of staff care regarding employee health, engagement and job satisfaction working on-site versus in a digital context. Against the background of inconsistent literature, which may be caused by the influence of specific sample characteristics or other third variables, we aim to systematically compare leadership effectiveness on-site versus digital. To exclude the influence of third variables, we conducted a laboratory experiment with one group working completely on-site, communicating via face-to-face contact with the leader, and one group working completely digital, communicating with the leader exclusively via video call.

With this approach we contribute to the literature in several ways. First, from a theoretical perspective, the study contributes to the deeper understanding of situational contingencies of leadership effectiveness in digital working environments. Up to now it remains an open question, whether health-oriented leadership can still be as effective in the digital working context as in more traditional working contexts with regular face-to-face contact. Our study thus adds to the existing literature on leadership effectiveness, and specifically the effectiveness of health-oriented leadership [4–8, 13, 14]. Second, the study contributes to the existing validity of the HoL concept as recommended by Rudolph et al. [15] by initially investigating health-oriented leadership in a fully digitalized working context. Moreover, as employees’ heart rate and heart rate variability are measured, the study follows the call for more objective measurements [2]. Third, from a methodological perspective, this is the first laboratory study in the context of health-oriented leadership. The design follows the call for more experimental studies in leadership research [16], reduces the risk for other confounding factors, and allows for causal conclusions. Fourth, from a practical perspective, as leaders and employees increasingly work from home, it is important to know in how far leadership is still effective when communicating only via digital media and how to advise leaders in terms of communication routines. Finally, the study adds to the current debate on leadership in the digital context and provides first empirical evidence for health-oriented leadership during the process of digitization [7, 12].

### Effects of health-oriented leadership on employee health and work-related attitudes

Previous research consistently showed relationships between leadership and employee health [17–20]. However, as Franke et al. suggested, general positive leadership behaviors such as LMX or transformational leadership may be too vague about health-specific leadership. Therefore, more health-specific leadership concepts came to the fore in leadership research. In 2014, Franke et al. introduced the concept of health-oriented leadership, which differentiates three related factors that contribute to employee health: [1] *leaders’ self care*, referring to leaders’ own health-related attitudes and behavior in terms of health-promoting self-leadership. Leaders’ self care is an important precondition for [2] *staff care*, comprising leaders’ health-promoting attitudes and behavior towards their employees (e.g., reduction of stressors and providing resources). Leaders’ self care and staff care both encourage and promote [3] *employees’ self care*, which describes followers’ dealing with their own health as well as health-promoting self-leadership. Studies show that health-specific leadership explains additional variance in health outcomes beyond transformational leadership and other generally constructive leadership behaviors [1, 21–23].

Empirical findings support the importance of health-oriented leadership and its components. Empirical evidence shows that health-oriented leadership contributes to follower health above and beyond other leadership behaviors, such as transformational leadership or LMX [1, 21, 22]. First, self care represents an internal resource for leaders and employees, which is related to a better general health-state, and less irritation, strain, health complaints, presentism and work-family conflicts [1, 13, 24–26]. Second, staff care represents an external resource for employees, which goes along with more resources and fewer stressors [1, 3]. Staff care is related to less irritation and strain, depression, and burnout. As staff care reduces employees’ risk factors for their physical well-being (e.g., stress and strain), staff care is not only related to a better mental health, but also to a better physical health [1, 2, 13, 25, 27–30]. Moreover, staff care is also positively related to employees’ work-related attitudes, such as job satisfaction, affective organizational commitment, engagement or job performance [2, 3, 24]. Given that studies on health-oriented leadership have already supported positive relationships not only with employee health, but also with their engagement and job satisfaction, we aim to replicate and extend these findings in the current study. Previous studies were often cross-sectional or used questionnaires with two points of measurement. In this case, interpretation in terms of causality is limited because third variables may influence employees’ perceptions of leadership and their own well-being. To exclude the influence of third variables and to enable causal conclusions, some recent studies on HoL utilized experimental studies [2, 21, 22, 31, 32]. However, realtime laboratory experiments that come close to real life settings are still missing. By testing the effectiveness of health-oriented leadership in a live experiment including objective health measurements, we aim to systematically control for staff care, to allow causal interpretations and to extend the existing validity of the HoL concept. Based on the existing literature, we expect the following:

#### Hypothesis 1

Staff care has *(a)* a negative effect on employees’ mental and physical exhaustion (i.e., lower HR and higher HRV-RMSSD), and *(b)* positive effects on employees’ engagement as well as *(c)* employees’ job satisfaction.

### The effectiveness of health-oriented leadership on-site compared to the digital working context

Health-oriented leadership aims at promoting employee health by reducing workplace-related stressors and providing resources [1]. However, the working context may be relevant for the effectiveness of HoL. Whereas it may be easier for leaders to effectively promote employee health and well-being while working on-site, it may be more difficult when working at home where contact is limited to digital media. Therefore, the question arises in how far staff care is effective in digital working environments. As we will argue, we assume that staff care may be less effective in digital working environments.

So far, research on leadership effectiveness in the digital context provides inconclusive findings. Whereas some studies show that leadership may be more effective in digital environments [4, 5], others suggest that leadership is particularly effective with regular face-to-face contact [6, 10, 11]. As there is no empirical evidence regarding health-oriented leadership yet, we analyze arguments for both sides in the following.

Some studies show that leadership may be even more effective in digital working environments. For example, a cross-sectional survey study by Golden and Veiga [5] showed that LMX was more effective and important for employees who predominantly work in the digital context. The authors conclude that employees value the perceived benefits of digital work and apt to reciprocate by being more satisfied, committed and increasing their performance. However, as most participants in this study were working in mixed forms, regular face-to-face contact was probably still given also when working digitally. A laboratory experiment by Purvanova and Bono [4] directly compared the effectiveness of transformational leadership for the face-to-face context and the digital context. Results showed that leadership was more effective for team performance in the digital condition than in the face-to-face condition. The authors assume that working teams are more dependent on the guidance of their leaders with regard to their joint performance due to uncertainties in digital working environments. Particularly in digital environments, leadership may improve coordination among team members and provide clear directions so that also team performance improves. Leaders may compensate a lack of direct contact among team members to ensure effective cooperation.

In contrast to Purvanona and Bono [4], who suggest that transformational leadership seems to be more important in digital working environments with regard to task fulfillment (i.e., team performance), a survey study by Jensen et al. [6] revealed that transformational leadership was more effective with face-to-face contact for relationship-oriented outcomes (i.e., mission valence). In line with media richness theory, the authors conclude that face-to-face contact represents a richer form of communication that enables leaders to provide multiple information cues, to offer individualized feedback in order to foster a shared understanding and meaning, and to personalize messages to employees. Therefore, the authors state that in terms of establishing high-quality relationships between leaders and employees, face-to-face contact is necessary to ‘get through’ to the employees.

It seems plausible that face-to-face contact is not only necessary for leaders to get through to employees, but that face-to-face contact is also necessary for leaders to perceive multiple information cues from employees in order to react appropriately. Therefore, it is conceivable that health-oriented leadership is more effective with in-person contact when it comes to more person-oriented and sensitive topics such as employee health. There is already evidence indicating that health-oriented leadership may be more effective with face-to-face contact. A recent interview study by Tautz et al. [11] identified five key challenges for staff care in digital environments: 1) *As social presence and interactions are reduced*, sensing the atmosphere and emotions within a team becomes difficult for leaders. While leaders get comprehensive impressions of employees during in-person meetings, they get less verbal and non-verbal cues in the digital context, so that the assessment of employee well-being is more challenging.

Less social presence also goes along with 2) *a lack of spontaneous and informal conversations*. According to Tautz et al. [11], employees rather disclose their personal needs, emotions and health issues in informal settings, for example, when spontaneously meeting up in the kitchen or while having lunch together. However, in digital settings, these opportunities are rare, which decreases employees’ disclosure and therewith leaders’ opportunities to react.

3) *Due to ICT hassles, communication quality may be reduced*. For example, when communication is interrupted by internet or system breakdowns, important verbal and nonverbal information may get lost and communication flow is obstructed. This makes it more difficult for leaders to perceive and react to employees’ warning signals [33].

4) *Also trust and bonding may be reduced*. As leaders and employees feel less close to each other in digital environments, the commitment for health-related recommendations may be reduced. For example, when leaders recommend to use a height adjustable desk or to finish work on time, it is difficult for them to monitor whether employees adhere to their recommendations and a health-promoting working style with spatial separation.

5) Finally, *leaders’ subjective responsibility for employee health decreases*, as it gets more difficult to influence employees’ health behavior and working environment. For example, their role model function is reduced as employees do not become aware of leaders’ health behavior with spatial separation. Due to spatial separation, also influencing employees’ working conditions is difficult for leaders, so that leaders feel less responsible.

Another interview study by Efimov et al. [10] revealed that the implementation of regular face-to-face meetings is important when teams are mainly working digitally. Leaders identified in-person meetings as highly relevant for health-oriented leadership, as they serve to discuss individual problems, employee health and well-being, or work-related issues in a trusting atmosphere.

In the digital context, important prerequisites for health-oriented leadership such as comprehensive impressions of employees, a trusting atmosphere and disclosure opportunities are less given while communication intensity and quality are reduced at the same time. Thus, it stands to reason that leaders’ efforts to engage in staff care may be less effective in digital working environments. As health is a very sensitive issue, it might be important to maintain regular face-to-face contact to ‘get through’ to the employees, so that the digital context is likely to decrease the effectiveness of staff care. In line with Jensen et al. [6], we hypothesize the following:

#### Hypothesis 2

Staff care is more effective for employees’ *(a)* mental and physical exhaustion (i.e., lower HR and higher HRV-RMSSD), *(b)* engagement, and *(c)* job satisfaction while working on-site than in the digital working context.

## Methods

### Sample and design

The study was carried out in spring 2022. A-priori power analysis using G*Power indicated *N* = 54 participants for a small effect size. As drop outs were to be expected, *N* = 71 participants took part in the experiment. Participants were military personnel enrolled at the university. Their participation was compensated with credits. After excluding one participant because of insufficient data and 10 participants that did not perceive the leader as health-promoting, the final sample consisted of *N* = 60 participants. Of these participants, 65% were male, 33.3% were female, and 1.7% divers. Most of the participants (75%) were between 18 and 25 years old (23.3% 26 to 35 years, 1.7% 36 to 45 years). All participants were experienced with digital work.

To test our hypotheses, we conducted a live experiment in a university laboratory with repeated measures. Participation was voluntary and anonymous. In order to create realistic working situations, a professional actor played the role of a health-oriented leader. In order to standardize the experimental condition, the actor was intensively trained before the final data collection. The leader (i.e., the actor) communicated and worked with employees either face-to-face while working on-site, or in a digital working environment via video call. Participants were randomly assigned to one of the groups (*N* = 30 face-to-face contact while working on-site; *N* = 30 digital working condition via video call). Simulating realistic conversations with real interaction partners instead of imagination as in previous vignette studies increases the validity of our study. In order to compare the effectiveness of staff care working on-site versus working digital, we utilized a 2 × 2 mixed design (within-subjects factor: no staff care [t1] vs. high staff care [t2]; between-subjects factor: working on-site vs. working digital).

### Procedure

Before the experiment started, participants got an informed consent and agreed to the participation and data processing. Participants were asked to take over the role of a trainee at the public relations department of the university. Employees’ work consisted in assisting in the writing process of press releases of their leader. The setting was introduced as follows: “*Please imagine that you started a new job as an employee in the press department of the university two weeks ago […] and are to assist your supervisor Mr. Lehmann. Mr. Lehmann is responsible for public relations and primarily takes care of the preparation of press distributions, press releases and press kits. […] You will be expected to assist Mr. Lehmann by performing minor support tasks such as proofreading. Mr. Lehmann has been on a business trip for the last two weeks. Therefore, you already received initial work assignments by e-mail. Today you will meet Mr. Lehmann in person in order to work with him on two short tasks during the next hour.*”.

Before employees met their leader in person, they received an e-Mail from him asking to correct a text for errors and to write a short final paragraph to round off the text before the meeting. Time limit was set to 10 min to complete this task in order to elicit some feeling of time pressure. While completing the task, heart rate and heart rate variability were measured through a chest strap. Afterwards, participants were asked about their current exhaustion, engagement and job satisfaction. These measures represent the pre-measurement for the within-subjects factor at t1 (i.e., no staff care condition). Up to this point, all participants worked under the same conditions.

After finishing the task and answering the questions, the leader either (1) physically entered the room, or (2) called employees via video call. This was to manipulate the working context as a between-subjects factor (i.e., on-site vs. digital working context). In both conditions, leaders and employees had the same conversation for about 15 min to get to know each other, to talk about the task and to talk about health issues. The health-oriented leader for example said “*It’s a somewhat stressful phase at the moment in which you are starting with us and we have a lot to do right now. Nevertheless, it is of course important to me that all my team members are doing well and that no one is overworking. So I do my best to take care of my employees’ health even in stressful phases, and I would also ask you to approach me if something is bothering you. […] What kind of person are you? Do you take a break when things get too much for you, or are you more the kind of person who needs support because of not noticing until it’s too late?*” or “*Especially now, after the last years of pandemic, many of us have reached our limits, and it was not always so easy to maintain the work-life balance due working from home. I realized that I had to actively take care of myself and do something for my health after being alone at home so much. I’ve been taking part in courses like this from the university’s occupational health management program ever since I’ve been able to do so again. I am currently taking part in the back school. Before the pandemic, I also tried the Strengths and Resources Training, which I can really recommend to you. How about your health activities?*”.

After the conversation, the leader assigned a second task to the employee. Again, the employee had to assist with a correction of a press text and to write a short final paragraph to finish the text. Time limit was again set to 10 min, and heart rate and heart rate variability were measured via chest strap during task completion. After finishing the task, participants again rated their current exhaustion, engagement and job satisfaction. These measures represent the post-measurement for the within-subjects factor at t2 (i.e., the high staff care condition). In order to check in how far the leader was perceived as health-oriented, participants afterwards rated the leaders’ health-orientation with three items.

After finishing the second task, leader and employees had a further conversation about the task and about their health-related issues. The conversation ended the experiment, and after finishing participants were informed about the study objectives.

### Measures

*Employee exhaustion.* Employees’ mental exhaustion was measured after participants worked on task 1 (t1) and task 2 (t2). Therefore, the subscale ‘personal burnout’ from the German version of the Copenhagen Psychosocial Questionnaire (COPSOQ) by N?bling et al. [34] with five items was used. Items were such as “*Right now*, *I feel emotionally exhausted*”. Items were rated on a five-point Likert scale from 1 = “*I totally disagree*” to 5 = “*I totally agree*”. Cronbach’s Alpha for t1 was α = 0.841 and α = 0.806 for t2.

Moreover, to measure employees’ physical exhaustion, their heart rate (HR) and heart rate variability (HRV) were measured using the Polar V800 heart rate monitor and the corresponding chest strap throughout the entire experiment. However, only the ten minutes during the processing of both tasks were analyzed. In Kubios HRV, two time samples were created for each participant: One sample (10 min) during task processing at t1 and one sample (10 min) during task processing at t2. To analyze the heart rate, average heart rate (beats per minute, bpm) was used. To analyze heart rate variability, RMSSD (in ms) was used. The RMSSD results from the variance of the temporal intervals between heartbeats and represents a measure of parasympathetic activity [35].

*Employee engagement*. Also employee engagement was measured after both tasks were completed. Employees’ engagement was measured with the ultra-short version of the Utrecht Work Engagement Scale [36] with three items, for example “*Right now, I am full of exuberant energy at work*”. Items were rated on a five-point Likert scale from 1 = “*I totally disagree*” to 5 = “*I totally agree*”. Cronbach’s Alpha was α = 0.816 for t1 and α = 0.875 for t2.

*Employee job satisfaction*. Similar to employee exhaustion and engagement, also job satisfaction was measured at t1 and t2. Job satisfaction was measured with a self-developed single item following the Job Diagnostic survey by Hackman & Oldham [37]. The item stated “*I am very satisfied with the current work situation”.* Items were rated on a five-point Likert scale from 1 = “*I totally disagree*” to 5 = “*I totally agree*”.

*Staff care*. To test whether the staff care manipulation was successful so that participants perceived the leader as health-oriented, staff care was measured after participants finished their second task (t2). We measured staff care with three items of the subscale ‘Staff Care’ from the Health-oriented Leadership scale by Franke et al. [1], for example “*This leader would immediately notice if something was wrong with my health*”. Items were rated on a five-point Likert scale from 1 = “*I totally disagree*” to 5 = “*I totally agree*”. For staff care, Cronbach’s Alpha was α = 0.711.

## Results

To assess the influence of staff care and its interaction with the working context on employee exhaustion, engagement and job satisfaction, we conducted a mixed repeated-measures ANOVA. Table 1 presents means and standard deviations for staff care (no staff care vs. high staff care) and the working context (on-site vs. digital). To ensure that direct effects of the IV on the DVs are not influenced by gender [38], we tested potential moderating effects. No moderating effects for gender were found.

**Table 1:** Means and standard deviations of the mixed ANOVA for employees’ mental exhaustion, physical exhaustion (HR, HRV), engagement and job satisfaction

	No staff care				High staff care						
	On-site		Digital		On-site		Digital				
	*M*	*SD*	*M*	*SD*	*M*	*SD*	*M*	*SD*	*F*	*p*	*η²*
Mental exhaustion	2.00	0.79	1.87	0.75	1.67	0.55	1.59	0.56	0.225	0.637	0.004
HR	81.61	11.71	82.21	11.70	74.53	30.52	79.66	11.09	0.724	0.398	0.012
HRV	38.95	19.17	45.90	22.81	42.30	29.54	50.89	24.38	0.138	0.711	0.002
Engagement	1.99	0.95	2.03	0.70	2.56	1.00	2.18	0.77	6.078	0.017	0.095
Job satisfaction	1.97	0.93	2.23	1.04	3.37	0.93	2.87	1.11	7.502	0.008	0.115

*Notes. N* = 60.

Hypothesis 1 proposed a negative effect of staff care on (a) employee exhaustion, as well as a positive effect of staff care on (b) employee engagement, and (c) employee job satisfaction. In line with our expectations, results showed a negative effect of staff care on employees’ mental exhaustion (*F* [1, 58] = 23.027, *p* < .001, η² = 0.284, *d* = 1.260). With regard to employees’ physical exhaustion, relationships failed to reach significance (HR: *F* [1, 58] = 3.261, *p* = .076, η² = 0.053, *d* = 0.473; HRV: *F* [1, 58] = 3.607, *p* = .063, η² = 0.059, *d* = 0.501). Moreover, results showed a positive effect of staff care on employee engagement (*F* [1, 58] = 17.240, *p* < .001, η² = 0.229, *d* = 1.090) and on their job satisfaction (*F* [1, 58] = 52.767, *p* < .001, η² = 0.476, *d* = 1.906). Hypothesis 1a is thus partially confirmed, while Hypotheses 1b and 1c are confirmed.

In Hypothesis 2, we expected that the effects of staff care on (a) employee exhaustion, (b) employee engagement, and (c) employee job satisfaction would be weaker in the digital working context. In contrast to our expectations, results of the mixed RM-ANOVA showed no significant interaction between staff care and the working context for employees’ mental (*F* [1, 58] = 0.225, *p* = .637, η² = 0.004, *d* = 0.127; Table 2) and physical exhaustion (HR: *F* [1, 58] = 0.724, *p* = .398, η² = 0.012, *d* = 0.220; HRV: *F* [1, 58] = 0.138, *p* = .711, η² = 0.002, *d* = 0.090; Table 2). There was no difference in the effectiveness of staff care working on-site versus working in the digital context for employee exhaustion. Hypothesis 2a is rejected.

**Table 2:** Results of the mixed RM-ANOVA for employees’ mental exhaustion, physical exhaustion (HR, HRV), engagement and job satisfaction

DV	source	*df*	*F*	*p*	*η²*	*d*
Mental exhaustion	Staff care	1	23.027	< 0.001	0.284	1.260
	Staff care x Group	1	0.225	0.637	0.004	0.127
HR	Staff care	1	3.261	0.076	0.053	0.473
	Staff care x Group	1	0.724	0.398	0.012	0.220
HRV	Staff care	1	3.507	0.063	0.059	0.501
	Staff care x Group	1	0.138	0.711	0.002	0.090
Engagement	Staff care	1	17.240	< 0.001	0.229	1.090
	Staff care x Group	1	6.078	0.017	0.095	0.648
Job satisfaction	Staff care	1	52.767	< 0.001	0.476	1.906
	Staff care x Group	1	7.502	0.008	0.115	0.721

*Notes. N* = 60.

**Fig. 1:**
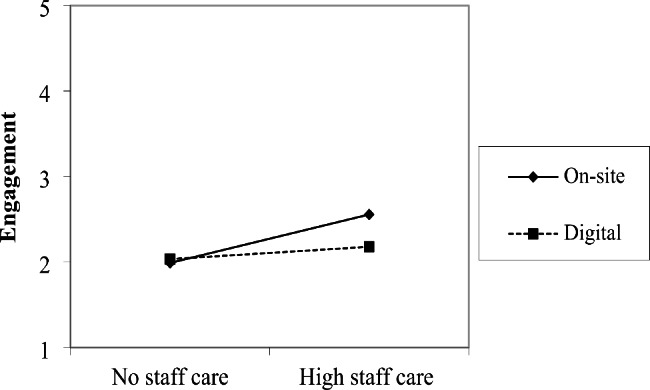
Interaction between staff care and working context for employees’ engagement

However, as depicted in Fig. 1, results of the mixed RM-ANOVA confirmed that the interaction effect of staff care and the working context was significant for employee engagement (*F* [1, 58] = 6.078, *p* < .05, η² = 0.095, *d* = 0.648; Table 2). In line with our expectations, the effect of staff care on employee engagement was weaker in the digital context than while working on-site. Hypothesis 2b is supported.

**Fig. 2:**
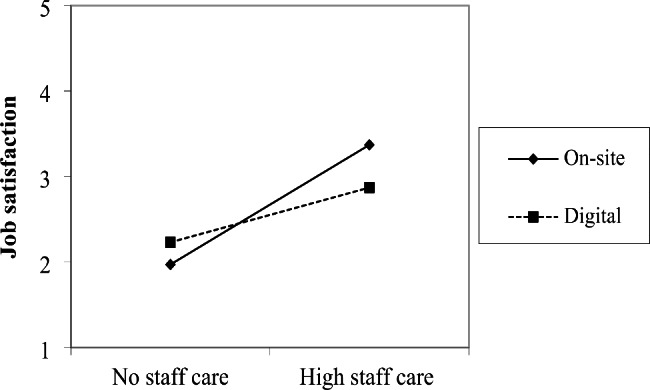
Interaction between staff care and working context for employees’ job satisfaction

As can be seen in Fig. 2, also the mixed RM-ANOVA for employee job satisfaction showed a significant interaction effect between staff care and the working context (*F* [1, 58] = 7.502, *p* < .01, η² = 0.115, *d* = 0.721; Table 2). In accordance with our expectations, the effect of staff care on employee job satisfaction was weaker in the digital context than while working on-site. Hypothesis 2c is confirmed.

## Discussion

Due to the proceeding digitization of work and increasing health risks for employees, the aim of the current study was to examine the effectiveness of health-oriented leadership in digital working environments compared to working on-site. We expected a negative effect of staff care on employee exhaustion, but a positive effect of staff care on employee engagement as well as job satisfaction. Moreover, we expected that the effects of staff care on employee exhaustion, engagement and job satisfaction are weaker in digital working environments than while working on-site. In line with these expectations, results revealed that staff care reduces employees’ physical and mental exhaustion, and promotes employee engagement and job satisfaction. Most importantly, results revealed that the effects of staff care on employee engagement and job satisfaction are weaker in digital working environments. However, staff care is equally effective for employee health (i.e., exhaustion) in digital working environments as while working on-site. These findings underline the notion that the effectiveness of staff care is mitigated in the digital context, particularly with regard to employee outcomes outside the health domain. In order to maintain the full effectiveness of staff care for employee health and work-related attitudes, regular face-to-face contact between leaders and employees seems to be essential.

The positive effects of staff care on employee exhaustion, engagement, and job satisfaction replicate and extend the positive relationships found in previous research on health-oriented leadership [1–3, 22, 28, 39]. In line with previous studies, staff care leads to a decrease in physical and mental exhaustion [1, 2, 29], and an increase in engagement [33] and job satisfaction [3, 24].

While previous studies mostly investigated the relationships between staff care and outcomes in survey studies, some studies even investigated causal effects using vignette and experimental designs [2, 22]. As a further step towards stronger validity, this study is the first to investigate staff care in a laboratory experiment with live conversations and real interactions between leaders and employees. In contrast to vignette experiments that depend on participants’ interpretations of the presented material and rather measure participants’ intentions for hypothetical outcomes, realistic experiments influence and measure actual behaviors and choices, affecting participants in real situations [16]. By actively manipulating leader behavior and the working context in real scenarios, this study is better able to assess the causal relationships between leadership, situational influences and employee outcomes as previous studies [16, 40]. Moreover, previous studies mostly investigated the effects of staff care on *subjective* health parameters [2, 29]. This study complements subjective measures by including *objective* health outcomes for the first time and shows positive effects on employees’ heart rate and heart rate variability, strengthening the validity of the HoL concept. Although the effects of staff care on employees’ physical health missed significance, the findings support the notion that staff care is an appropriate behavior to foster not only subjective, but also objective employee health and therewith both employees’ *mental* and *physical* health.

While previous studies investigated staff care in rather traditional working environments with regular face-to-face contact, hardly anything is known about its effects in digital working environments [11, 41]. Therefore, the experiment initially compared the effectiveness of staff care while working on-site to working in a digital context. Surprisingly, results revealed that the effectiveness of staff care for employees’ exhaustion is not mitigated in digital working environments. Instead, staff care is equally effective for employees’ physical and mental health in digital working environments and while working on-site. Although communication quality and intensity are reduced and a trusting atmosphere is less given in digital working environments [10, 11], leaders can also effectively promote their employees’ health without face-to-face contact. This is an important finding, as the primary goal of health-specific leadership is to foster employee health and well-being [1]. Health-oriented leaders can achieve this goal also in the digital working context, so that staff care is still worth to display when leaders and employees do not have any face-to-face contact at all. However, it is possible that we did not find a difference for exhaustion because a baseline effect may have occurred. As baseline exhaustion levels at t1 were rather low in both groups (on-site: *M*_*t1*_ = 2.00; digital: *M*_*t1*_ = 1.87), it was probably difficult for the leader to reduce employees’ exhaustion even more. Hence, no differences in the effectiveness of staff care could have been detected between working on-site and working digitally. Another explanation is that the time lag between both measurement points was too short for changes in exhaustion. Future studies could induce stress using validated stress manipulations in the beginning to enable stress reduction and use longer time lags between the measurements in order to enable a detection of potential differences in leadership effectiveness for employee exhaustion.

However, according to our expectations, the positive effects of staff care on employee engagement and job satisfaction are mitigated in digital working environments. Staff care is less effective for employees’ engagement and job satisfaction when leaders and employees solely communicate via video conference. This finding underlines the risks for the effectiveness of healthy leadership, as it seems to be harder for health-oriented leaders to promote employees above and beyond their health in the digital context. In the literature it is assumed that the positive effects of healthy leadership on employee outcomes outside the health domain are at least partly explained by an increase in employee health [1, 13, 20]. However, as important corresponding and important influencing factors such as social proximity and bonding between leaders and employees are less given [11, 12], fostering employee engagement and job satisfaction as a by-product of health-promotion is less effective in digital environments without face-to-face contact. In line with Jensen et al. [6], our results indicate that it is necessary for leaders to maintain regular face-to-face contact in order to get through to employees, so that staff care can unfold its full effectiveness to promote not only employee health, but also their engagement and job satisfaction.

### Theoretical implications

The present study highlights the notion that staff care represents an important workplace resource not only for employees’ exhaustion, but also for their engagement and job satisfaction in both digital working environments and while working on-site. By displaying staff care, leaders support their employees by reducing work-related demands and providing resources, which fosters their health, their engagement, and their satisfaction [1, 3]. By initially replicating the positive effects of staff care in a live experiment with real interactions between leaders and employees, we were able to influence and measure actual behaviors, choices and feelings of participants in real situations [16]. Moreover, by showing positive effects of staff care on employees’ *objective* health in terms of their heart rate and heart rate variability, our results provide first tentative evidence that staff care also affects employees’ objective physical health. Thus, the study follows the call for more experimental leadership research and more objective measurements [2, 16]. The study thus contributes to the existing validity of the HoL concept and underlines the notion that staff care not only affects subjective health and well-being, but also objective health outcomes.

Moreover, it is a new insight that staff care is equally effective for employee health in digital environments as while working on-site, but that the effectiveness regarding employee engagement and job satisfaction is mitigated in digital working environments. Up to now, empirical research regarding leadership effectiveness in digital environments remains scarce [10–12]. By initially uncovering the digital working context as an influencing factor on the relationship between staff care and employee outcomes, the current study further extends the validity of the HoL concept. In line with Media Richness Theory [42] and in line with the findings of Jensen et al. [6], the study shows that staff care is still effective for employee health in digital environments, but that rich communication (i.e., face-to-face contact) is necessary in order to unfold its full effectiveness also for outcomes above and beyond employee health (i.e., engagement and job satisfaction).

### Limitations and recommendations for future research

The current study has some limitations that should be considered. Although the experimental scenarios come close to reality, participants were aware that they were in an experimental setting for a limited period of time. This limited time period might have been too short to uncover leadership effects, which usually develop over weeks or months. This may have led to an underestimation of the effects. In order to validate the current findings and to capture complex leader-follower relationships, future research should aim to replicate our findings in the field. However, it is important to note that we found positive effects even within this short period of time. This leads to the assumption that the positive effects of staff care may be even stronger in reality.

This may particularly apply to the findings regarding employees’ heart rate and heart rate variability, which failed to reach significance in the current study. It is remarkable that differences in objective health values have emerged in this short period of time at all, and it seems plausible that staff care has even stronger effects on employees’ physical health when conversations and relationships are more intensified. Therefore, future studies should investigate staff care, particularly with regard to objective health outcomes, in further experiments with longer lasting interactions between leaders and employees and with repeated measurements.

Moreover, due to live interactions between the leader and employees, conversation content slightly differed between participants. As the leader had real in-person conversations with employees (i.e., the participants), we were not able to estimate all possible answers of the employees to the leaders’ questions. Thus, it was not possible to fully standardize the experimental condition, which may have led to varying perceptions in the amount of staff care. To ensure consistent quality of the experimental condition, we chose a qualified actor who was also experienced in improvisation theatre and the actor was intensively trained before starting the experiment. To ensure consistent quality over the course of trials, the authors regularly monitored the actors’ performance.

### Practical implications

This study underlines the notion that leaders can serve as an important workplace resource for employee health, engagement and job satisfaction. As results show, staff care is equally effective for employee exhaustion in the digital context as while working on-site. However, the effectiveness of staff care for employee outcomes outside the health domain (i.e., engagement and job satisfaction) is mitigated in digital working environments. The current findings suggest that in order to foster not only employee health, but also employee engagement and job satisfaction, leaders should keep regular face-to-face contact also when work predominantly takes part in the digital context. Face-to-face contact and in-person meetings enable informal conversations, disclosure opportunities, and the development of trust and bonding, which may help health-oriented leaders to foster employee engagement and job satisfaction above and beyond employee health [11, 12]. Therefore, organizations should provide leaders and their teams with regular possibilities of in-person contact, as video conferences cannot replace the effects of real face-to-face interactions. For example, it is important that organizations provide offices, conference rooms and particularly travel funds, so that leaders can meet their employees in person. These in-person meetings should take place on a regular basis in order to foster commitment and binding between leaders and employees.

Moreover, organizations should make leaders aware of their responsibilities for employee health and well-being also in the digital working context when they are spatially separated. Therefore, organizations should invest in leadership trainings and health-oriented leadership interventions [43, 44]. These leadership trainings should particularly focus on how to maintain and foster employee health and other work-related attitudes also over distance and aside from face-to-face contact.

## Conclusion

The current study is the first to compare the effectiveness of health-oriented leadership between working on-site and the digital working context. The study thus contributes to the deeper understanding of leadership effectiveness in digital working environments and uncovers corresponding risks for health-oriented leadership. Results replicate previous findings of positive relationships between staff care and employee health, engagement and job satisfaction. Moreover, the study initially provides objective evidence of positive effects on employee health in terms of their heart rate and heart rate variability. Most importantly, results revealed that staff care is equally effective for employee health in the digital context as while working on-site, but that the effectiveness with regard to employee engagement and job satisfaction is mitigated in the digital working context. These findings suggest that in order to foster employees’ engagement and job satisfaction, organizations must provide leaders and employees with the required framework conditions to maintain face-to-face contact on a regular basis, also when work predominantly takes part in the digital context.

## Data Availability

The dataset is available from the corresponding author on reasonable request.
